# Case mix at the European Institute of Oncology: first report of the Tumour Registry, 2000–2002

**DOI:** 10.3332/ecancer.2009.149

**Published:** 2009-10-21

**Authors:** E Botteri, S Iodice, P Maisonneuve, M Alfieri, N Burzoni, L Manghi, M Martinetti, B Montanari, E Albertazzi, B Bazolli, N Rotmensz

**Affiliations:** Division of Epidemiology and Biostatistics, European Institute of Oncology, via Ripamonti 435, Milan, Italy

## Abstract

**Introduction:**

An institutional and centralized hospital-based tumour registry (TR) is the ideal supporting tool for the organization and management of clinical data in a comprehensive cancer centre. The purpose of this paper is to describe the development of the TR at the European Institute of Oncology (IEO), Milan, Italy, from its origin to its current applications.

**Material and methods:**

After a series of meetings with members of administrative, clinical, research and informatics departments, the TR was activated in March 2006 with the aim of collecting data on all the individuals referred to the institute, with or at risk of developing a tumour. It was implemented on an Oracle™-based interface. A minimum dataset of variables was defined and data collection was divided into four forms, which together gather all the relevant data on patients, tumours, treatments and subsequent events.

**Results:**

After a six-month pilot period, which involved the training of the tumour registrars, adjustments to the structure of the registry, development of a data quality control procedure and finalization of the operative protocol, since September 2006 the data collection has been fully operative. Five registrars have been chronologically entering data of all individuals who visited the IEO for the first time since 1 January 2000. As of March 2009, data on 69,637 individuals and 43,567 tumours has been reviewed, recoded and registered in the TR. Twenty-two per cent of the tumours (*n*=9578) were first invasive primaries, diagnosed and treated in the IEO; the most common sites were breast (*n*=4972), lung (*n*=627), intestines (*n*=479) and prostate (*n*=376).

**Conclusion:**

The IEO TR has been proven functional and reliable in monitoring the activity of the hospital, allowing extraction of data from any subpopulation with characteristics of interest. The structured and centralized TR represents an important tool for our research-oriented institution.

## Introduction

The European Institute of Oncology (IEO) aims at excellence in the prevention, diagnosis and treatment of cancer through high-level clinical and scientific research. Since its opening in 1994, the number of individuals presenting for the first time to the IEO has increased year by year, reaching 38,500 in 2008. Thus, the need of a well-structured and centralized institutional database has become essential. Furthermore, to optimize the use of the great quantity of information, some obstacles typical of the organization and management of hospital data had to be overcome, such as the presence of many non-standardized databases, duplication of information and absence of centralized follow-up. For these reasons, the IEO tumour registry (TR) was planned, designed and finally activated in 2006.

The purposes of a hospital-based TR are to support the administration of the hospital, back up the clinical and scientific research and, above all, serve the needs of the patient [1]. In fact a TR can provide immediate reports on the activity of the hospital, document the cancer burden borne by the hospital for specific periods of time, provide background information useful for the design of clinical studies, extract data of any subpopulation with characteristics of interest and organize a centralized follow-up of patients, avoiding duplication of information and useless contact between the hospital and the patients.

The aims of the present paper are to illustrate the development of the IEO TR, from its origin to its current applications, and to describe the case-mix of our institute. We will report data from its first three years of activity, including data on all individuals presenting to the institute between 2000 and 2002, already having or being at risk of developing a tumour.

## Material and methods

### Brief history

The TR project was developed in many successive phases, starting with a series of meetings and discussions on ‘who’ an ‘what’ to include. A group of experts, consisting of physicians, information technology (IT) specialists, data managers, administrative personnel and biostatisticians had regularly met for almost two years to discuss the format and the data to be collected. An important part of the planning was also dedicated to visiting and contacting other hospital-based cancer registries, in order to learn from already established realities. Thereafter, the database was designed and tested. It was decided to implement the TR using an Oracle™-based interface. The implementation, completed in February 2006, was managed in the Division of Epidemiology and Biostatistics, with the help of the IT division. The next phase involved the selection and training of dedicated data registrars. The project finally got off the ground in March 2006, and at present five dedicated persons are working full time on entering the data. Data quality control and data management are carried out by two data managers and two biostatisticians.

### Eligibility criteria

We agreed to collect information on all individuals presenting at the IEO since its opening, either with a tumour or at risk of developing one. We also decided to collect information on patients with previous invasive tumours, either treated at this institute or elsewhere. In order to gain entry in the TR, patients must have: (1) the IEO unique identification number, assigned to the individual at the first visit; and (2) at least one medical report (i.e., pathological report or diagnostic examination) accessible from the institute’s intranet.

### Information collected

A minimum dataset of variables was defined, to establish the best compromise between synthesis and informativeness. Data collection is divided into four forms: on the first form personal data (i.e. sex, date of birth) and information on survival (i.e. date of last contact, date of last visit, vital status, cause of death) are recorded. The type of record is registered in this form. The following types of record are assigned to each individual.

1. ***Visit:*** a healthy individual comes to the IEO for either a visit or a genetic counselling.
2. ***Anamnesis:*** the patient, at the moment free of disease, reports on a tumour diagnosed in the past and already treated and cured elsewhere.
3. ***Diagnosis:*** diagnosis of tumour is made at the IEO and the patient decides to be treated and followed up elsewhere.
4. ***Second opinion:*** the patient or the patient’s parents come to the IEO and ask for an opinion on a diagnosis and/or a treatment proposed elsewhere.
5. ***Long:*** the patient receives at least one treatment at the IEO.

Detailed information on the patient’s tumour(s) (i.e. date of diagnosis, morphology, topography, TNM staging, ordinal number of the tumour for multiple tumours) is recorded on the second form, together with some epidemiological information (i.e. familiarity, height and weight at diagnosis and smoking habits). The third form is dedicated to the treatment strategy, where every therapy is classified as administered or proposed. The fourth catalogues, chronologically, all following events, tracing the whole history of the disease.

It is important to note that the amount of data collected will vary according to the type of record: the completion of all four forms only occurs for patients coded as ‘Long’. For patients coded as ‘Anamnesis’, ‘Diagnosis’ or ‘Second opinion’ only the personal data form together with date of diagnosis, place of diagnosis, the IEO histopathological identification code (if any), topography and morphology are registered. For patients coded as ‘Visit’ only the personal data form is completed.

Invasive and *in situ* tumours are always collected, whereas benign tumours or negative histologies are collected only if the patient undergoes a major surgical procedure in the IEO. Examples of benign tumours/negative histologies we collect are: adenoma of the thyroid after thyroidectomy, fibroadenoma after quadrantectomy and negative histology after hysterectomy.

**Figure 1:**
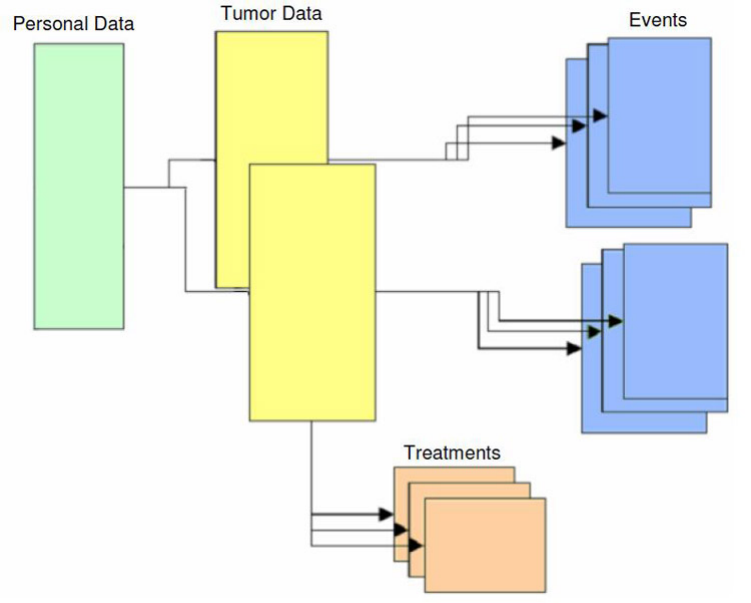
The structure of the IEO tumour registry

The structure of the TR is represented in Figure 1. The patient is the basis of the structure. Zero, one or more tumours can be linked to the patient. Zero, one or more treatments and events can be linked to each tumour.

Systematized Nomenclature of Medicine-Clinical Terms (SNOMED CT) [2] is used to classify tumours according to topography and morphology except for haematopoietic tumours, for which we use the World Health Organization (WHO) ICD-10 classification [3]. Clinical and pathological staging is based on the TNM system [4], whether the fifth or the sixth edition according to the date of diagnosis. Cause of death is based on the WHO ICD-10 classification [3].

### Sources of data, data entry and quality control

The IEO TR data entry is completely paperless, as all the information derives from computerized sources: the IEO patients’ administrative database (personal information is automatically visible on the personal data form), medical reports accessible on the IEO intranet, online databases (surgery, laboratory medicine, pathology, etc.) and patients’ clinical dossier, which are all digitalized and accessible online.

Five data registrars work full time on data entry. Each one has specialized in a particular tumour or set of tumours in order to achieve specific knowledge and abilities. Data quality control and data management are carried out by two data managers and two biostatisticians. A data manager randomly reviews data entered by a registrar, and the two discuss potential mistakes. A field was created in which notes regarding which cases are revised by a data manager and which are not inserted. Furthermore, in order to clarify any doubt on specific cases, a medical doctor was chosen from each division as a referent to address questions to.

## Results

A six-month pilot period (March to August 2006) was necessary to train the registrars, adjust the structure of the registry, develop a data quality control procedure and edit the operative protocol. Since September 2006, the data entry has been running at top speed. We started by entering individuals who presented for the first time to the IEO in the year 2000 in a sequential fashion. By March 2009, 69,637 individuals had been entered in the TR, corresponding to individuals who came for the first time to the IEO between 2000 and 2002, not necessarily presenting with a tumour. In Table 1, some characteristics of these individuals are reported. Approximately, one out of four was classified as ‘Long’, as he/she received at least one treatment in the IEO. Only 1.6% of individuals were 19 years old or less, as our institute does not treat paediatric tumours.

Overall, 39,480 individuals out of 69,637 had one or more tumours. Information on a total of 43,567 tumours was collected, with an average of 1.1 tumours per person. Frequencies by site, malignancy, place of diagnosis and place of surgery are reported in Table 2.

In Table 3, we reported only the first invasive primary tumours, diagnosed and treated in the IEO (*n*=9578), which represent the typical subgroup of tumours used for epidemiological retrospective studies. Breast was the most frequent tumour site by far (*n*=4972), followed by lung (*n*=627), intestines (*n*=479) and prostate (*n*=376). In order to show an example of the reports that can be easily obtained from the TR, we included some additional information for the most frequent tumour sites: breast (women only), colorectal, lung, non-Hodgkin lymphoma, ovary, prostate, sarcoma, skin melanoma and tongue (Appendices A–I].

Finally, we calculated that, on average, it took about ten minutes to enter a case, that is six cases per hour. The time varied depending especially on the type of record: 22 minutes for a long case, two minutes for a visit and nine minutes for other types of record. By the end of 2009, data of all individuals who referred to the IEO for the first time in the years 2000–4 will be collected, for a total of about 125,000 individuals and 80,000 tumours.

## Discussion

This paper presents the first three years of activity of the IEO TR, specifically data on all individuals presenting for the first time to the institute in the years 2000–2, already having or being at risk of developing a tumour. This first analysis of the TR data demonstrated that the registry can support the administration in monitoring the hospital activity, be a supporting tool for the clinical practice as well as for epidemiological/basic research, play a key role in the production of publications on any subpopulation with characteristics of interest and, above all, improve patient management and knowledge of the disease.

Regarding information on events and survival, although patients in the TR had a theoretically adequate follow-up, with a median of six years, we preferred not to report any result, as we have not performed a detailed analysis of the quality of the follow-up yet. By now, vital status (dead/alive) and events are based on passive follow-up, with an accuracy depending on the site or other characteristics of the tumours. For example, since our institute produces many publications in breast cancer field, follow-up information on patients suffering from that tumour is updated more frequently compared to patients with other tumours. Survival analyses on different tumours might have been incomparable. By 2010, after a detailed analysis of the follow-up information collected in our institute, an active centralized follow-up will be organized and started.

Although it was decided that researchers or clinicians would not have direct access to the TR, we have established a system to collect and answer requests. Data extrapolation and analysis are managed in the Division of Epidemiology and Biostatistics.

A recent paper entitled ‘Analysis of local and regional recurrences in breast cancer after conservative surgery’ represents the first study conducted on the data of the TR [5]. It consisted of a multistage analysis of local, regional and distant recurrences, performed on data of 2784 women treated for early breast cancer by quadrantectomy and whole breast irradiation at the IEO. The authors concluded that ‘local and regional recurrences after breast-conserving surgery are rare events. They are markers of tumour aggressiveness and indicators of an increased likelihood of distant metastases’.

As shown in this paper, we can easily monitor the activity of the IEO and extrapolate data from any subpopulation with characteristics of interest. We strongly believe that a well-structured and centralized TR is a necessary tool for our, and in fact any, institution where research is a fundamental part of its activity.

## Figures and Tables

**Table 1:**
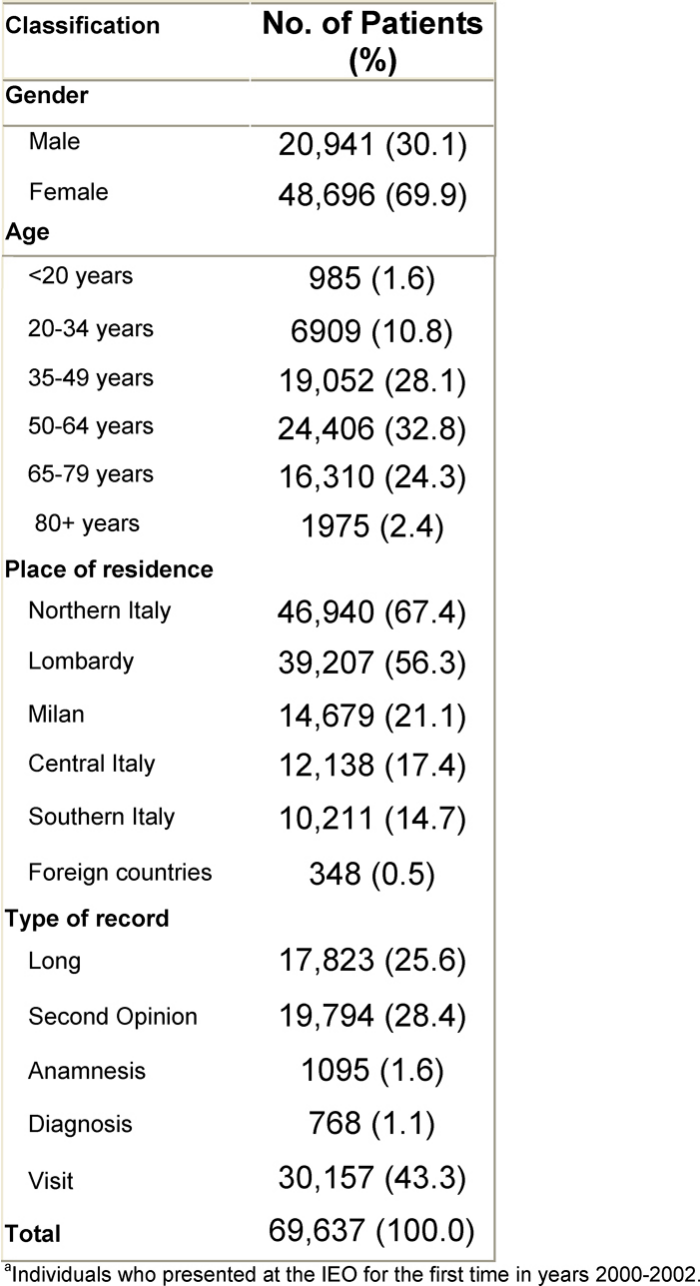
Characteristics of individuals^a^

**Table 2:**
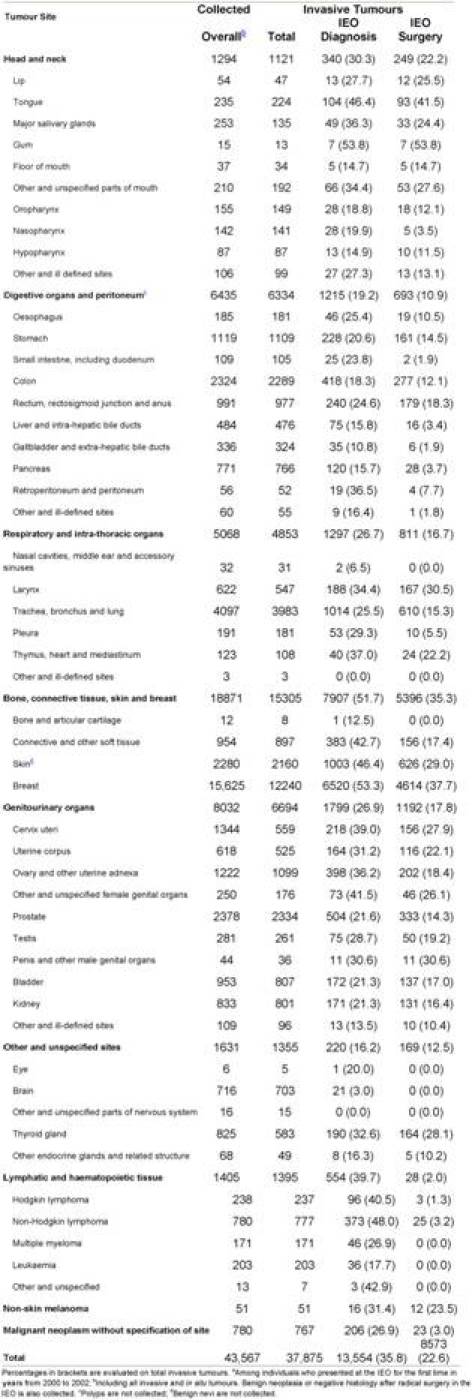
Tumours by site^a^

**Table 3:**
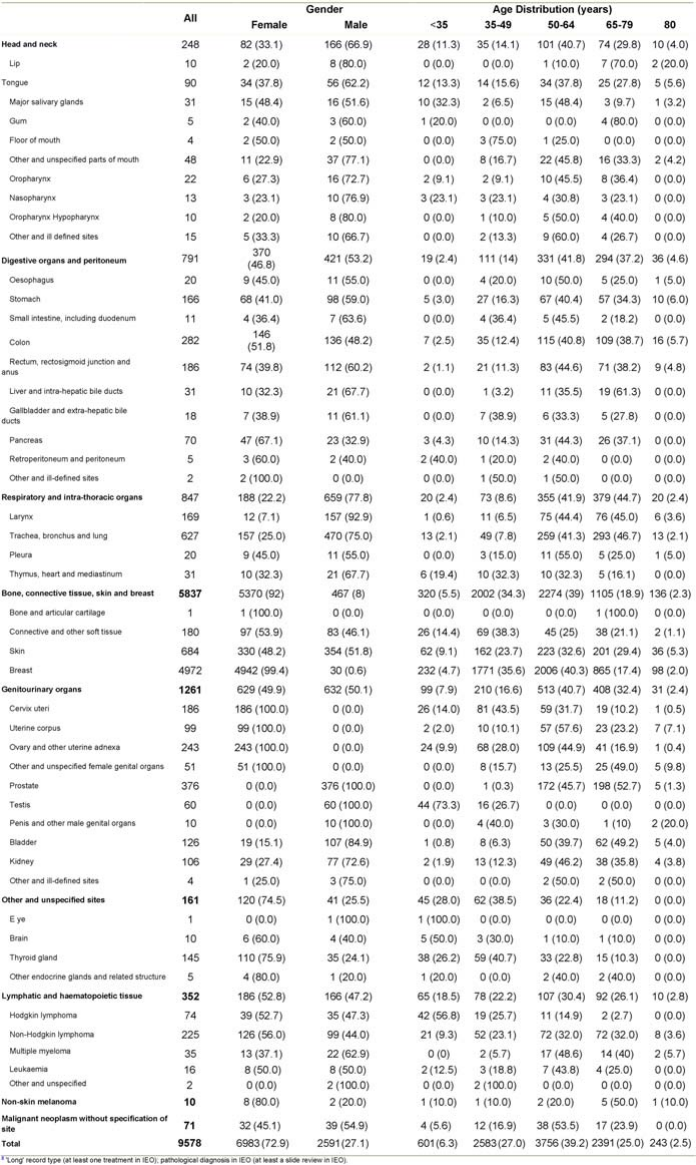
First invasive primaries diagnosed and treated in the IEO^a^ by site, gender and age

**Table A1:**
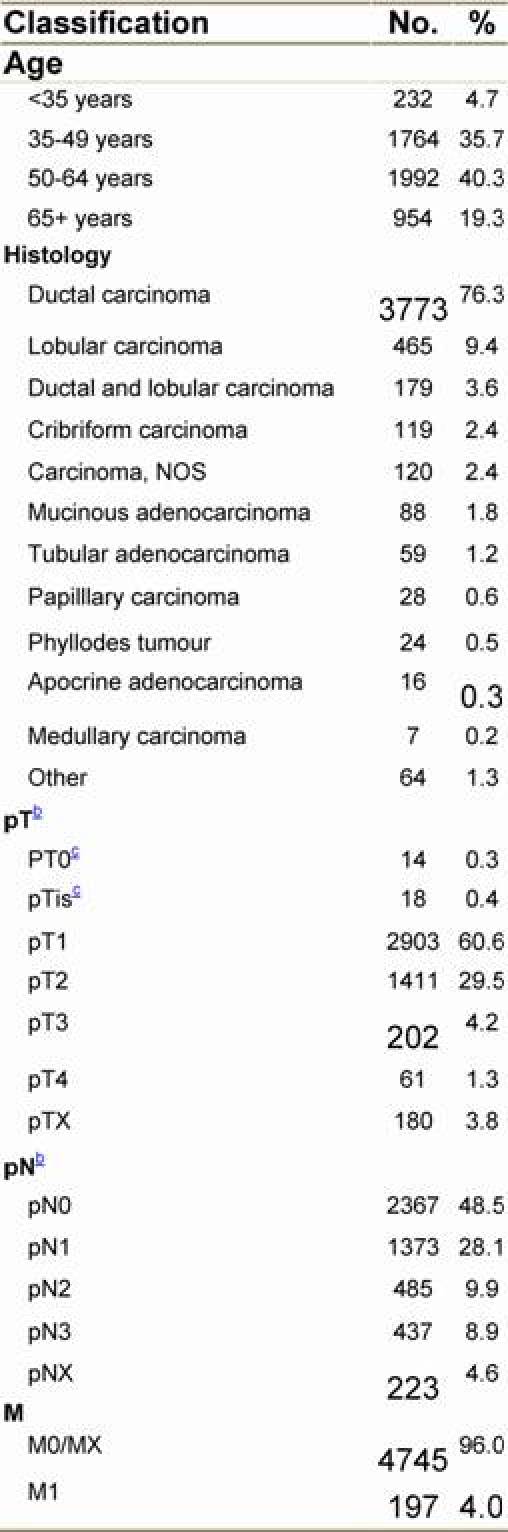
Characteristics at diagnosis

**Table A2:**
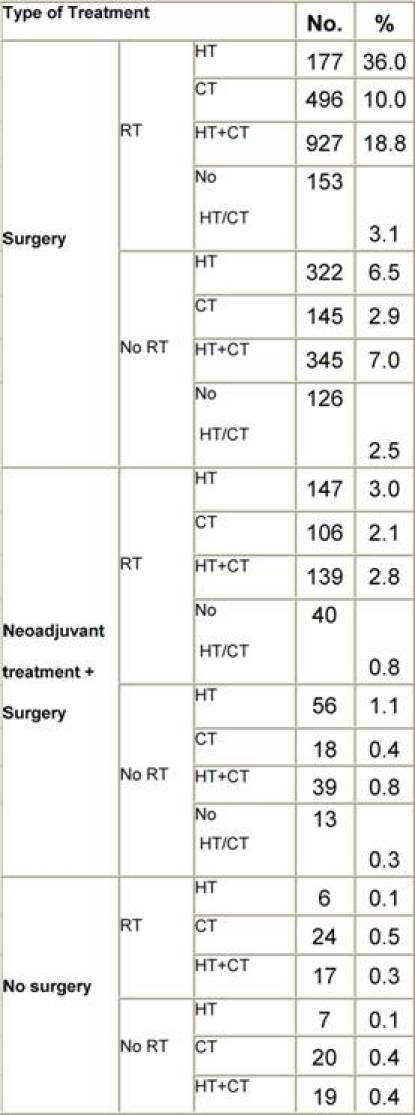
Treatment strategy

**Figure A1:**
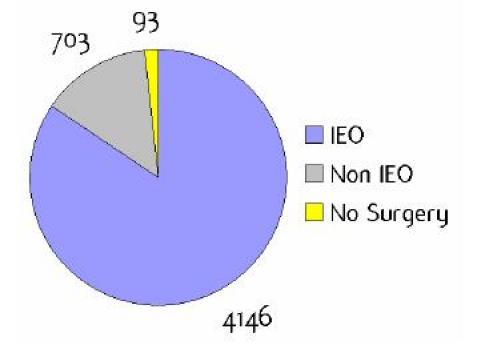
Place of surgery

**Fig A2:**
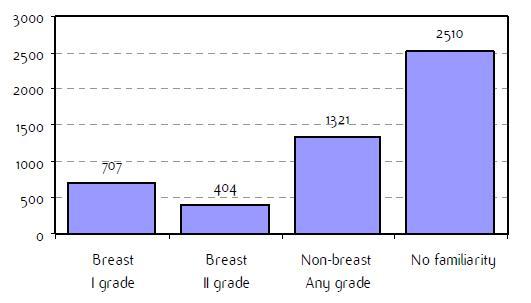
Familiarity for breast and non-breast cancer^b^

**Table B1:**
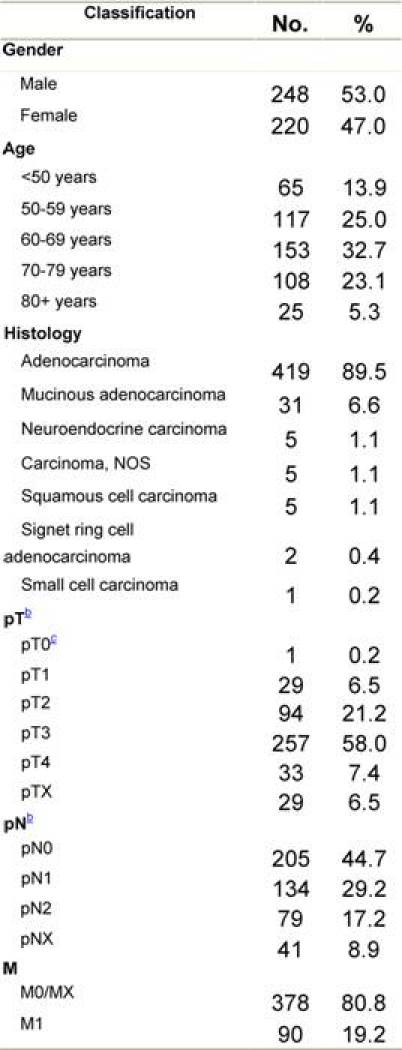
Characteristics at diagnosis

**Table B2:**
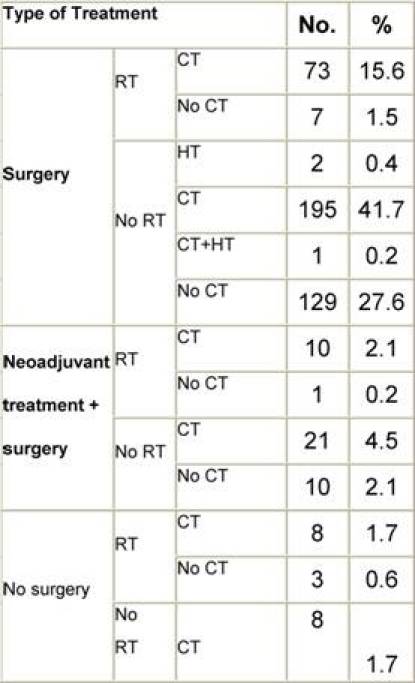
Treatment strategy

**Figure B1:**
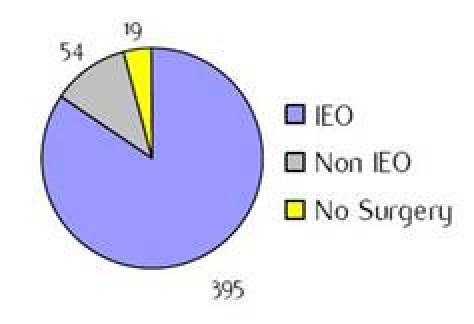
Place of surgery

**Figure B2:**
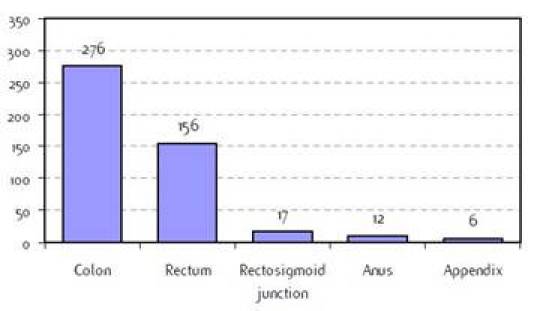
Specific tumour site

**Table C1:**
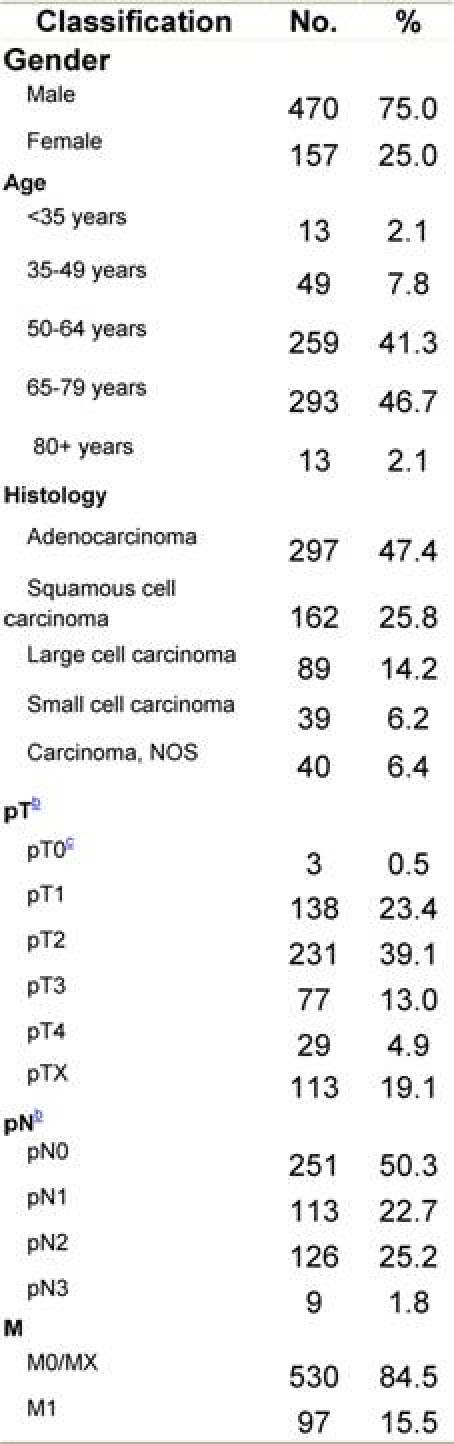
Characteristics at diagnosis

**Table C2:**
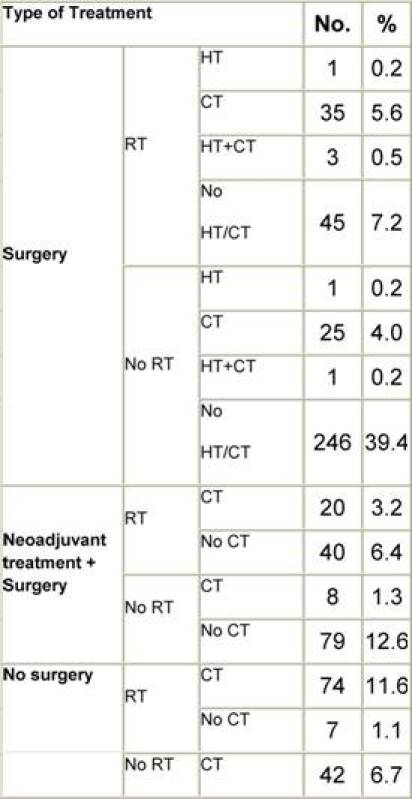
Treatment strategy

**Figure C1:**
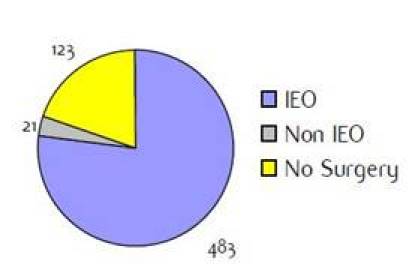
Place of surgery

**Figure C2:**
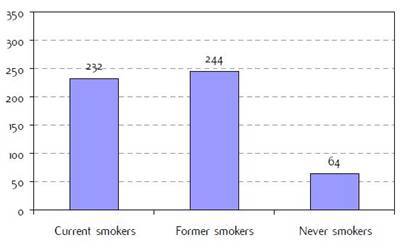
Smoking status at diagnosis^b^

**Table D1:**
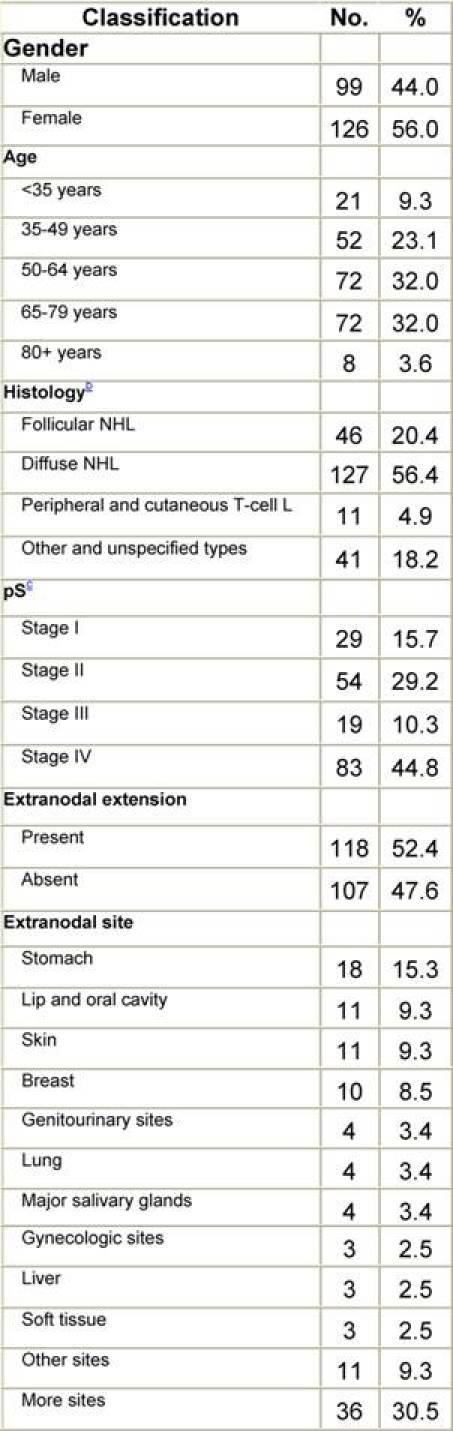
Characteristics at diagnosis

**Table D2:**
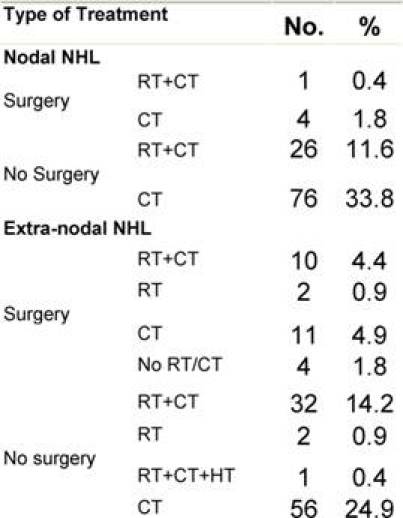
Treatment strategy

**Figure D1:**
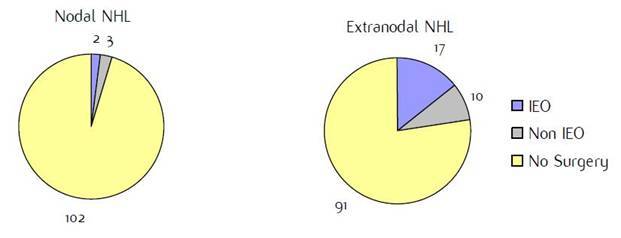
Place of surgery

**Table E1:**
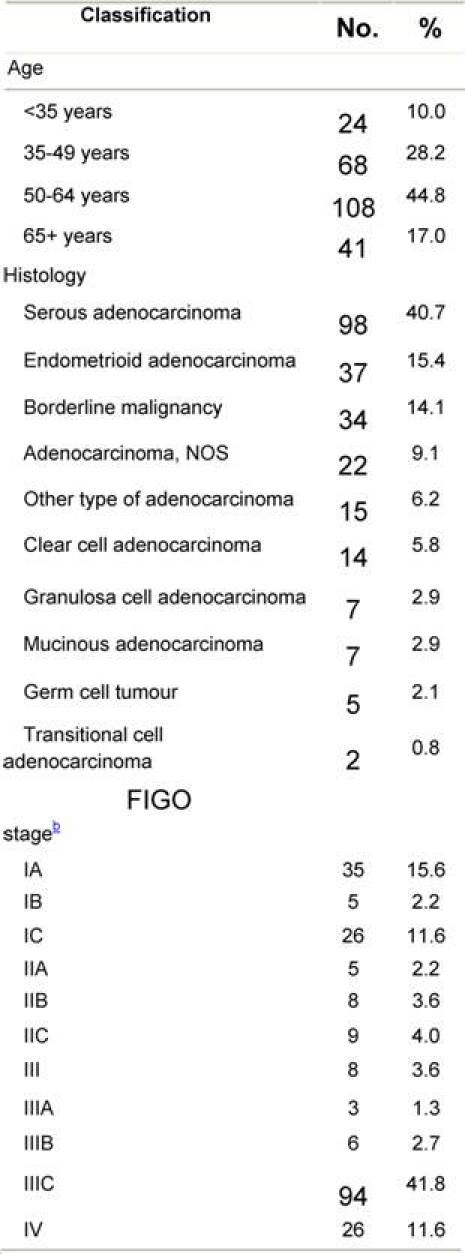
Characteristics at diagnosis

**Table E2:**
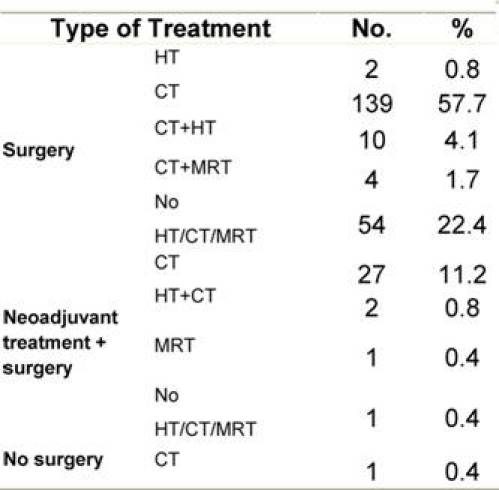
Treatment strategy

**Figure E1:**
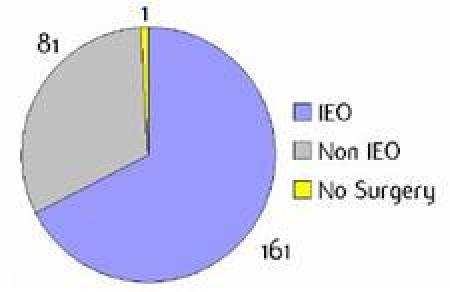
Place of surgery

**Figure E2:**
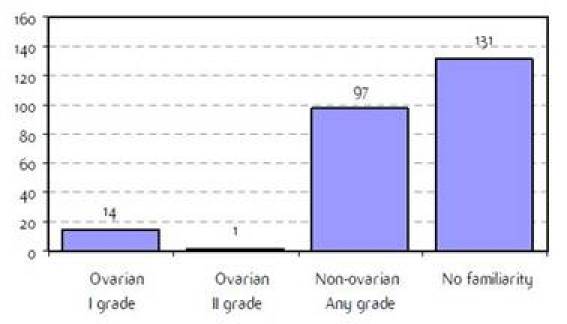
Familiarity for ovarian and non-ovarian cancer

**Table F1:**
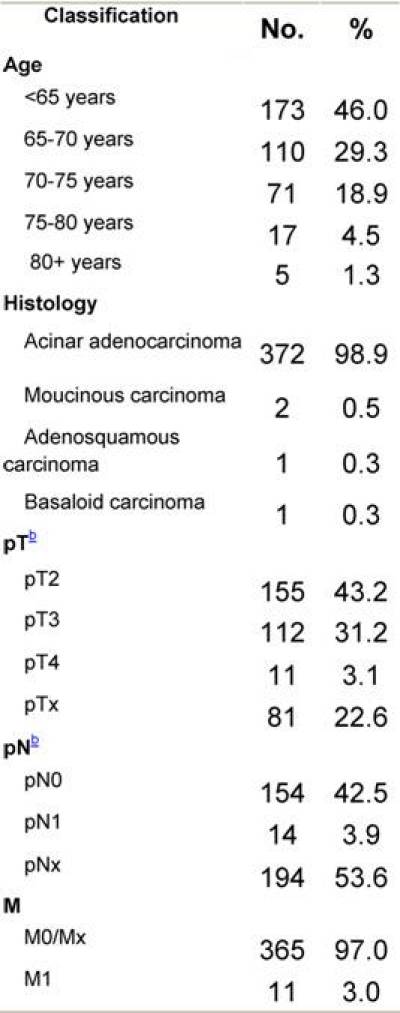
Characteristics at diagnosis

**Table F2:**
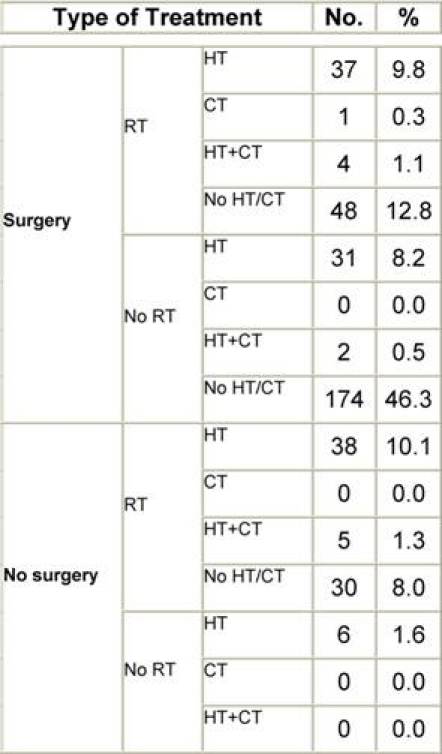
Treatment strategy

**Figure F1:**
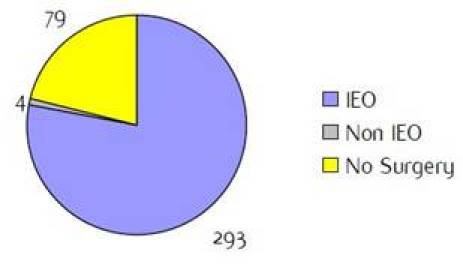
Place of surgery

**Figure F2:**
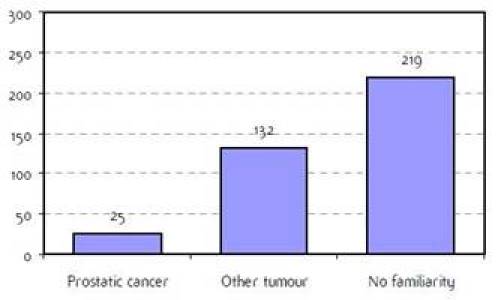
Familiarity for prostatic and non-prostatic cancer

**Table G1:**
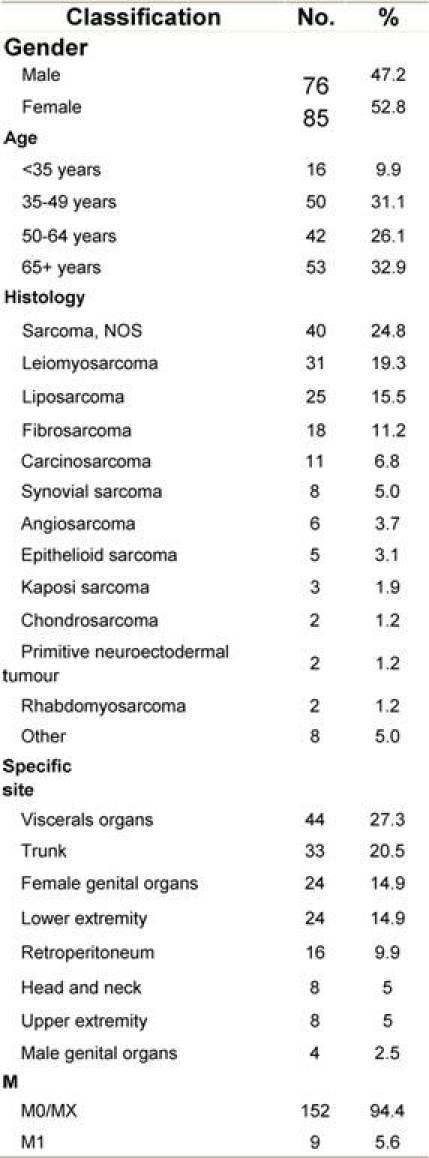
Characteristics at diagnosis

**Table G2:**
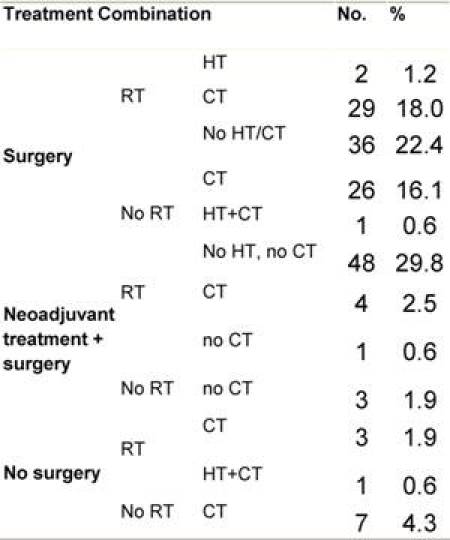
Treatment strategy

**Figure G1:**
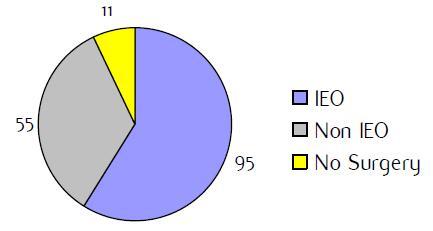
Place of surgery

**Table H1:**
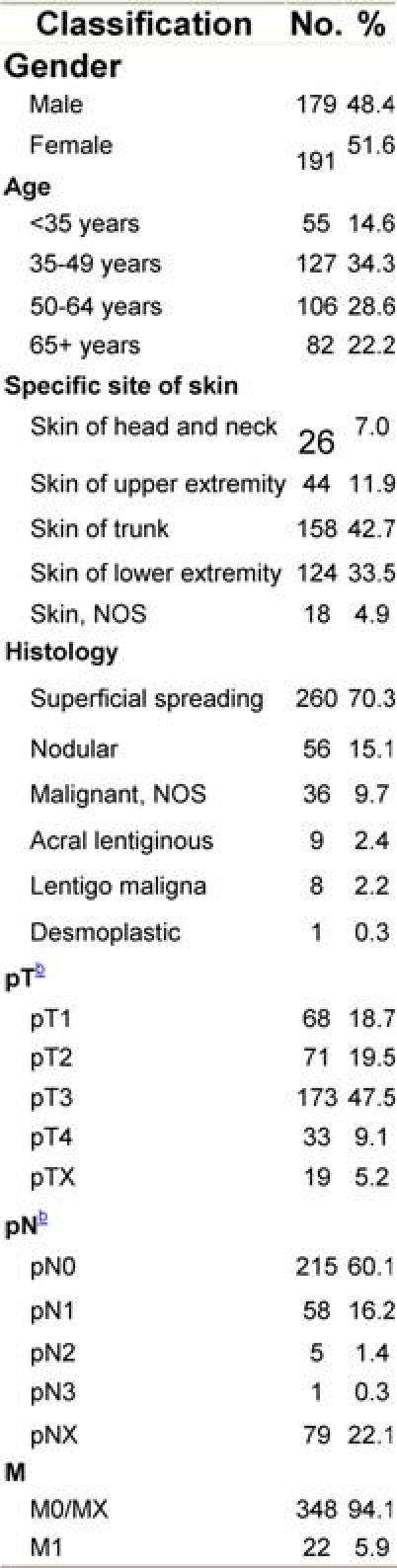
Characteristics at diagnosis

**Table H2:**
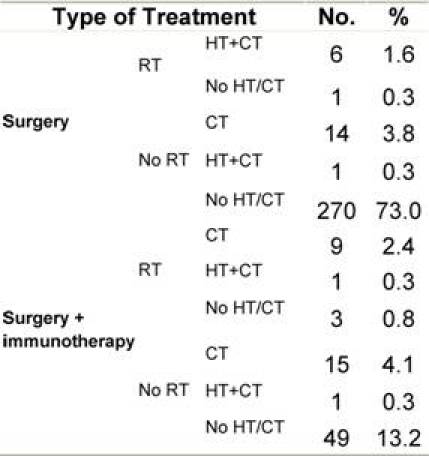
Treatment strategy

**Figure H1:**
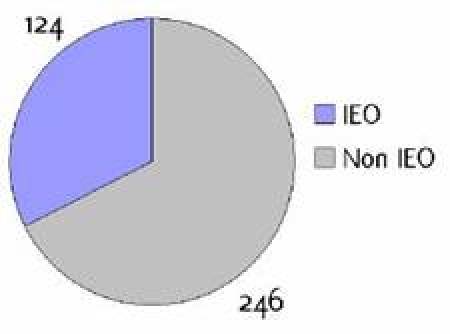
Place of surgery

**Figure H2:**
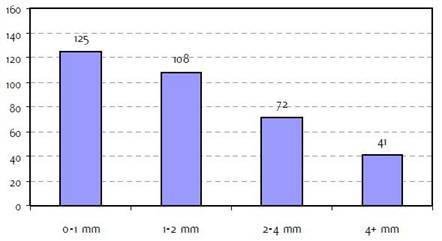
Breslow thickness ^b^

**Table I1:**
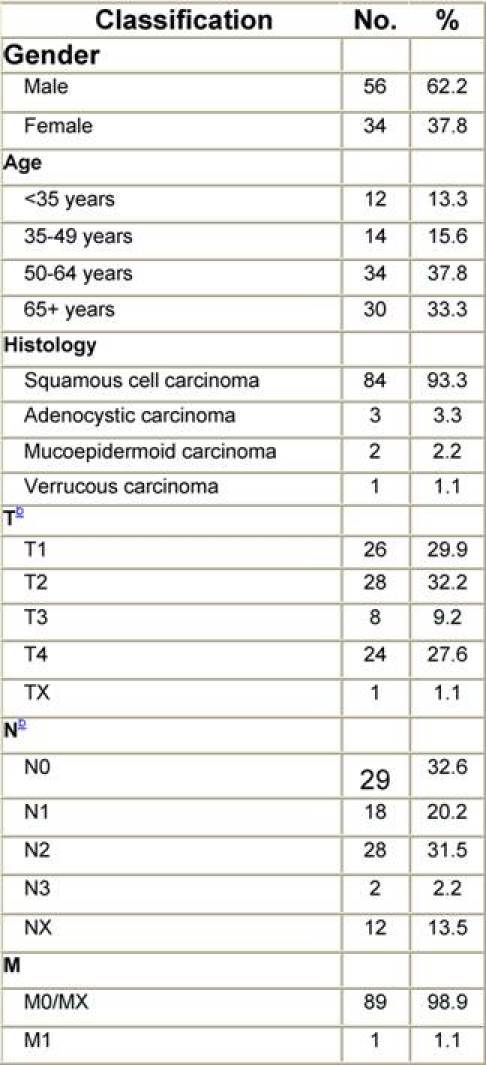
Characteristics at diagnosis

**Table I2:**
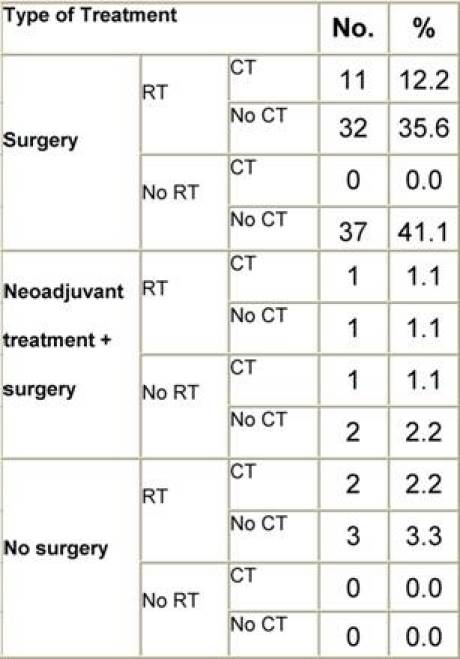
Treatment strategy

**Figure I1:**
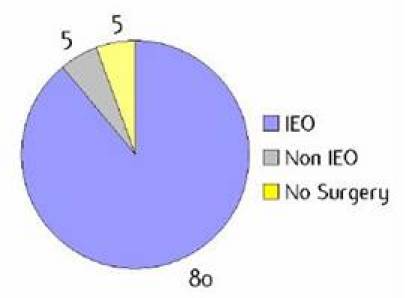
Place of surgery

**Figure I2:**
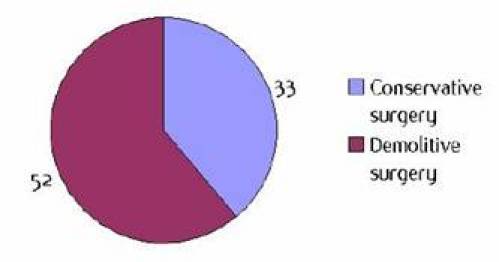
Type of surgery

## Appendices

### Appendix A: Characteristics broken down by tumour type: breast cancer (women only)

^a^‘Long’ record type (at least one treatment in IEO); pathological diagnosis in IEO (at least a slide review in IEO); ^b^Information is missing for some patients; ^c^No evidence of primary tumour after neoadjuvant treatment. RT: Radiotherapy; HT: hormonotherapy; CT: chemotherapy and/or monoclonal antibody. NOS: not otherwise specified.

### Appendix B: Characteristics broken down by tumour type: colorectal cancer (468 first invasive primaries treated and diagnosed in IEO^a^)

^a^‘Long’ record type (at least one treatment in IEO); pathological diagnosis in IEO (at least a slide review in IEO); ^b^Information is missing for some patients; ^c^No evidence of primary tumour after neoadjuvant treatment. RT: radiotherapy; HT: hormonotherapy; CT: chemotherapy and/or monoclonal antibody; NOS: not otherwise specified

### Appendix C: Characteristics broken down by tumour type: lung cancer (627 first invasive primaries treated and diagnosed in IEO^a^)

^a^‘Long’ record type (at least one treatment in IEO); pathological diagnosis in IEO (at least a slide review in IEO); ^b^information is missing for some patients; ^c^No evidence of primary tumour after neoadjuvant treatment. RT: radiotherapy; HT: hormonotherapy; CT: chemotherapy and/or monoclonal antibody.

### Appendix D: Characteristics broken down by tumour type: non-Hodgkin lymphoma (225 first invasive primaries treated and diagnosed in IEO^a^)

^a^‘Long’ record type (at least one treatment in IEO); pathological diagnosis in IEO (at least a slide review in IEO); ^b^WHO classification applied; ^c^Information is missing for some patients. pS: pathological stage. RT: Radiotherapy; HT: hormonotherapy; CT: chemotherapy and/or monoclonal antibody. NHL: non-Hodgkin lymphoma; L: lymphoma.

### Appendix E: Characteristics broken down by tumour type: ovarian cancer (241 first invasive primaries diagnosed and treated in IEO^a^)

^a^‘Long’ record type (at least one treatment in IEO); pathological diagnosis in IEO (at least a slide review in IEO); ^b^Information is missing for some patients. HT: Hormonotherapy; CT: chemotherapy and/or monoclonal antibody; MRT: metabolic radiotherapy; NOS: not otherwise specified.

### Appendix F: Characteristics broken down by tumour type: prostate (376 first invasive primaries treated and diagnosed in IEO^a^)

^a^‘Long’ record type (at least one treatment in IEO); pathological diagnosis in IEO (at least a slide review in IEO); ^b^information is missing for some patients; RT: radiotherapy; HT: hormonotherapy; CT: chemotherapy and/or monoclonal antibody. NOS: not otherwise specified.

### Appendix G: Characteristics broken down by tumour type: sarcoma (161 first invasive primaries treated and diagnosed in IEO^a^)

^a^‘Long’ record type (at least one treatment in IEO); pathological diagnosis in IEO (at least a slide review in IEO); ^b^information is missing for some patients. RT: Radiotherapy; HT: hormonotherapy; CT: chemotherapy and/or monoclonal antibody; NOS: not otherwise specified.

### Appendix H: Characteristics broken down by tumour type: skin melanoma (370 first invasive primaries diagnosed and treated in IEO^a^)

^a^‘Long’ record type (at least one treatment in IEO); pathological diagnosis in IEO (at least a slide review in IEO); ^b^information is missing for some patients. RT: Radiotherapy; HT: hormonotherapy; CT: chemotherapy and/or monoclonal antibody. NOS: not otherwise specified.

### Appendix I: Characteristics broken down by tumour type: tongue (90 first invasive primaries diagnosed and treated in IEO^a^)

^a^‘Long’ record type (at least one treatment in IEO); pathological diagnosis in IEO (at least a slide review in IEO); ^b^information is missing for some patients; RT: radiotherapy; HT: hormonotherapy; CT: chemotherapy and/or monoclonal antibody.
